# Mitochondrial Gene Diversity and Host Specificity of *Isospora* in Passerine Birds

**DOI:** 10.3389/fvets.2022.847030

**Published:** 2022-06-29

**Authors:** Steven V. Kubiski, Carmel Witte, Jennifer A. Burchell, Dylan Conradson, Alexandra Zmuda, Alberto Rodriguez Barbon, Jose G. Vilches-Moure, Stephen A. Felt, Bruce A. Rideout

**Affiliations:** ^1^Disease Investigations, Conservation Science and Wildlife Health, San Diego Zoo Wildlife Alliance, San Diego, CA, United States; ^2^College of Veterinary Medicine, Washington State University, Pullman, WA, United States; ^3^Poznan University of Medical Sciences, Poznan, Poland; ^4^Veterinary Department, Jersey Zoo, Trinity, Jersey; ^5^Department of Comparative Medicine, Stanford University, Stanford, CA, United States

**Keywords:** *Isospora*, coccidia, passerine, cytochrome oxidase, zoological

## Abstract

*Isospora* infections are common in both wild and captive passerine species. Many bird species have been shown to have co-evolved with a particular species of *Isospora*. Disease can range from subclinical to severe and fatal, making infection and transmission of this parasite a concern for birds under managed care, particularly in institutions housing endangered species for breeding and reintroduction purposes. Whether birds in mixed-species enclosures represent a risk factor for severe isosporiasis due to infection with non-host-adapted strains is of concern for institutions managing these populations. To begin answering this question, we sought to characterize the host-specificity of *Isospora* spp. in a large number of passerine birds *via* retrospective sequencing of mitochondrial gene cytochrome *c* oxidase subunit I (COI). Despite outliers, *Isospora* sequences largely grouped by host species and/or host family. Additional research is warranted into the degree of interspecies transmission and host-switching of *Isospora* parasites, and risk factors for the development of severe disease in passerine birds.

## Introduction

The genus *Isospora* refers to apicomplexan coccidia of the family Eimeriidae. The group has had a fairly confusing history taxonomically, with deeper work on host and parasite genetics yielding clarification and adoption of nomenclature change (1, 2). The current convention is that *Isospora* spp. refers to avian parasites within Eimeriidae, “*Atoxoplasma*” is an obsolete term referring to the previous characterization of *Toxoplasma*-like parasites in the blood, and *Cystoisospora* be reserved for similar parasites but infecting mammals (1).

*Isospora* spp. are considered ubiquitous, and like other members of the Eimeriidae, are thought to be host-specific, likely co-evolved with their definitive hosts (2–4). Experimental infections with *Eimeria* species across different taxa including frogs, mice, and birds, demonstrate that a successful infection is genus- or species-specific (4–7). There is, however, evidence for interspecies transmission and mixed species infections in natural settings (2, 8). Further, it is unknown if an exposure to new strains of *Isospora* spp. presents a higher risk for developing severe disease.

Interspecies transmission is possible in managed-care settings, where mixing diverse avian species increases the risk of exposure to non-host-adapted parasites. Compared with natural settings, this provides greater opportunities for “species-jumping,” or sporadic opportunistic interspecies transmission, as well as “host-switching”—longer term host–pathogen relationship reflecting an adaptive, evolutionary process (9, 10). While infections can be incidental, higher population densities and mixed-species aviaries make clinical isosporiasis a concern due to the potential for stress and high environmental parasite burdens. Additionally, reports of severe isosporiasis in birds from conservation breeding programs have resulted in a heightened awareness of birds' exposure to non-host-adapted parasites (11–13). This is of particular concern for rare and endangered species, as well as those bred for release into the wild that may expose threatened free-ranging populations to a novel pathogen.

This retrospective survey had two objectives. The first was to demonstrate the diversity of *Isospora* spp. among a wide range of avian taxa in mixed-species environments. The second was to identify whether these parasite lineages exhibit host-specificity, as the mitochondrial cytochrome *c* oxidase subunit I (COI) gene has been shown to be a reliable marker for assessing species diversity within the Eimeriidae (14, 15).

## Materials and Methods

### Study Birds and Samples

Samples included in this study came from three different groups of passerine birds. First, archived, frozen tissues (−70°C) available from necropsied passerine birds with diagnoses of protozoal infections between 1996 and 2020 were identified from the San Diego Zoo Wildlife Alliance (SDZWA). Search terms included, *Isospora, Atoxoplasma*, coccidia, protozoa, and their variations, as well as mention of intracellular parasites seen in peripheral blood smears. Birds determined to be infected with protozoa other than *Isospora* (such as *Hemoproteus, Plasmodium*, and *Leukocytozoon*), were not included. Archived, frozen tissues in the resulting search were obtained by routine collection at post-mortem and submitted to San Diego Zoo Wildlife Alliance's Molecular Diagnostic Laboratory for Polymerase Chain Reaction (PCR). Necropsied birds in this group included captive-hatched, wild-hatched, and native wild birds found dead, with representative taxa from North and South America, Europe, Africa, and Asia.

The second group of samples included fecal samples opportunistically collected from 20 live birds from 1996 to 2020 *via* an approved SDZWA biomaterials request.

The third group included samples from birds from external institutions that all died with severe systemic isosporiasis. One from a recently wild caught colony of 13 European starlings from the San Francisco Bay area housed at Stanford University, and tissues from 16 blue-crowned laughingthrushes from Jersey Zoo (UK).

### Deoxyribonucleic Acid Isolation

Deoxyribonucleic acid (DNA) was extracted from previously frozen feces and tissue samples. For the feces extraction, the Qiagen DNA Powersoil Kit (Valencia, CA) was utilized according to the manufacturer's protocol with the addition of a pretreatment step of four freeze-thaw cycles (3 min in liquid nitrogen followed by 3 min at 95°C). Tissue extraction was done with the Qiagen Dneasy Blood and Tissue Kit (Valencia, CA) following the manufacturer's tissue extraction protocol. Each sample DNA was eluted at 100 μl. The resultant DNA was stored short-term at −20 and −80°C long-term.

### Cytochrome *c* Oxidase Subunit I Polymerase Chain Reaction Assay

Polymerase chain reaction (PCR) assays were performed using the Eppendorf Mastercycler Pro S thermocycling system (Hauppauge, NY). The general procedure was taken from Ogedengbe et al. (14) with forward primer Cocci_COI_For – 5′-GGTTCAGGTGTTGGTTGGAC-3′ and reverse primer Cocci_COI_Rev −5′-AATCCAATAACCGCACCAAG-3′ for the COI target gene. Each 25 μl reaction contained the following reagents: 12.5 μl of 2× MyTaq HS Mix (Bioline, Memphis, TN), 400 nM of each primer (IDT, San Diego, CA), 2 μl of the tissue extracted DNA, and 4 μl of the fecal extracted DNA was added to each reaction. Each sample was run with a known positive control, negative extraction control, and PCR negative control. Controls were used to detect the success of the PCR as well as any contamination that might have occurred during the extraction or PCR set up. For the COI assay, the following PCR parameters used were as follows: 96°C for 5 min, (94°C for 20 s, 55°C for 30 s, 72°C for 90 s) ×40, 72°C for 10 min. The amplicons were run on a 0.9% agarose gel stained with 2.5% ethidium bromide. Bands of the expected size of ~780 bp were isolated and subsequently gel-extracted using the EMD Millipore Ultrafree™-DA (Millipore, Billerica, MA).

### Cloning and Sequencing

The gel-extracted amplicons were subsequently cloned following the manufacturer's protocol using Invitrogen's TOPO TA Cloning kit (Carlsbad, CA) with TOPO 10 chemically competent cells. The transformed cells were grown on carbenicillin-inoculated LB agar plates overnight at 37°C. Two colonies per each clone were picked, inoculated in LB broth with carbenicillin (100 μg/ml), and allowed to propagate overnight at 37°C in a shaking incubator set at 200 rpm. The broth culture was extracted using Zymo Zyppy Plasmid Miniprep Kit (Irvine, CA) according to the manufacturer's protocol. The plasmid-extracted DNA was checked for quantity and quality using the BioTek Synergy Take3 plate (Winooski, VT). The plasmid DNA was submitted to Eton Bioscience (San Diego, CA) and Retrogen (San Diego, CA) for Sanger sequencing with universal primers T7 promoter and M13 reverse.

### Analysis

The analysis of generated sequences was performed using Geneious 10.2.6 (Newark, NJ). Raw sequencing files were uploaded and each direction of the cloned sequences were trimmed using the trim vectors function [Univec (high sensitivity)] option. The trim primers function was simultaneously used in choosing the appropriate primer according to the assay that was run. Default parameters were utilized for the trim vector function and trim primers function. Next, each sequence was pairwise aligned utilizing the Geneious alignment program and a consensus alignment was performed. The program Basic Local Alignment Search Tool nucleotide blast (BLASTn) was utilized from the National Center for Biotechnology Information (NCBI) database to determine sequences of similarity and speciate the sample sequence alignments. The query length as well as the percent identity and percent query coverage were used to evaluate the significance of the BLAST hits of the sequenced sample. Sequences were aligned in Geneious Prime 2020.2.2 using MUSCLE 3.8.425 (16). Phylogeny was inferred using a maximum likelihood method with the Tamura–Nei (17) model in MEGA-X (18, 19). The model selection function for optimal nucleotide substitution was utilized, and maximum likelihood trees were generated with 500 bootstrap replicates. A neighbor-joining consensus tree was also generated using Geneious tree builder, with the Tamura–Nei substitution model, using the COI gene from *Toxoplasma gondii* as the outgroup and bootstrap percentages over 1,000 replicates.

## Results

The total number of generated sequences was 181, representing tissues from 136 birds and 43 different species (Table 1). All sequences have been deposited in GenBank (accession numbers OL999103–OL999283; Supplementary Table 1). Genetic distances across all COI sequences ranged from 92.3 to 100% with a mean of 95.7%. Identical sequences occurred only between the tissues from birds of the same species, including blue-crowned laughingthrush, white-rumped shama, crested oropendola, European starling, superb starling, and golden-breasted starling.

**Table 1:** Avian host species and associated tissue samples represented by *Isospora* sequences.

**Species (scientific name)**	**Number individuals tested (*N* = 136)**	**Tissues tested** **(Total = 181)**
Black-naped oriole (*Oriolus chinensis*)	1	GI (1)
Black-throated laughingthrush (*Pterorhinus chinensis*)	1	Liver (1), spleen (1)
Blue-crowned laughingthrush (*Pterorhinus courtoisi*)	20	GI (4), heart (1), liver (11), lung (2), muscle (2), pool (1), spleen (5)
Blue-faced honeyeater (*Entomyzon cyanotis*)	1	GI (1)
Cape sparrow (*Passer melanurus*)	1	GI (1)
Chestnut-backed scimitar–babbler (*Pomatorhinus montanus*)	1	GI (1), lung (1)
Chinese hwamei (*Garrulax canorus*)	7	GI (7), liver (2), lung (3), spleen (1)
Collared finchbill (*Spizixos semitorques*)	2	GI (2), liver (1), lung (1)
Crested oropendola (*Psarocolius decumanus*)	2	GI (2), liver (1), lung (1), spleen (1)
Eastern green-and-gold tanager (*Tangara schrankii*)	1	Spleen (1)
Emerald starling (*Lamprotornis iris*)	6	GI (3), liver (2), lung (1), spleen (2)
European goldfinch (*Carduelis carduelis*)	3	GI (2), liver (3), lung (1), skin (2)
European starling (*Sturnus vulgaris*)	13	GI (2), lung (11)
Fairy-bluebird (*Irena puella*)	4	GI (2), liver (1), lung (1), spleen (2)
Fawn-breasted bowerbird (*Chlamydera cerviniventris*)	3	GI (3)
Golden-breasted starling (*Lamprotornis regius*)	8	Blood (1), GI (2), liver (3), lung (1), Spleen (2)
Golden-hooded tanager (*Tangara larvata*)	1	GI (1)
Gouldian finch (*Chloebia gouldiae*)	1	GI (1)
Greater necklaced laughingthrush (*Pterorhinus pectoralis*)	1	Liver (1)
Grosbeak starling (*Scissirostrum dubium*)	2	GI (2), liver (1), lung (1)
House sparrow (*Passer domesticus*)	5	GI (5), liver (1)
Lesser goldfinch (*Spinus psaltria*)	1	GI (1)
Northern mockingbird (*Mimus polyglottos*)	1	GI (1)
Oriole warbler (*Hypergerus atriceps*)	1	GI (1)
Palila (*Loxioides bailleui*)	2	GI (2)
Purple grenadier (*Uraeginthus ianthinogaster*)	1	GI (1)
Purple honeycreeper (*Cyanerpes caeruleus*)	1	GI (1)
Raggiana bird-of-paradise (*Paradisaea raggiana*)	6	GI (6)
Raven (*Corvus corax*)	1	GI (1)
Red-billed leiothrix (*Leiothrix lutea*)	1	GI (1)
Scarlet-faced liocichla (*Liocichla ripponi*)	1	GI (1)
Snowy-crowned robin-chat (*Cossypha niveicapilla*)	1	GI (1)
Sociable weaver (*Philetairus socius*)	4	GI (4)
Spotted towhee (*Pipilo maculatus*)	1	Lung (1)
Superb starling (*Lamprotornis superbus*)	11	GI (7), liver (6), lung (4), spleen (1)
Swallow tanager (*Tersina viridis*)	1	GI (1)
Taveta golden weaver (*Ploceus castaneiceps*)	1	GI (1)
Western crow (*Corvus brachyrhynchos*)	3	GI (3)
Western tanager (*Piranga ludoviciana*)	2	GI (2)
White-headed buffalo weaver (*Dinemellia dinemelli*)	2	GI (2)
White-rumped shama (*Copsychus malabaricus*)	8	GI (6), heart (1), liver (3), lung (3), skin (2)
White-throated laughingthrush (*Pterorhinus albogularis*)	1	Liver (1)
Yellow-crowned bishop (*Euplectes afer*)	1	GI (1)

*GI, gastrointestinal*.

**Figure 1:**
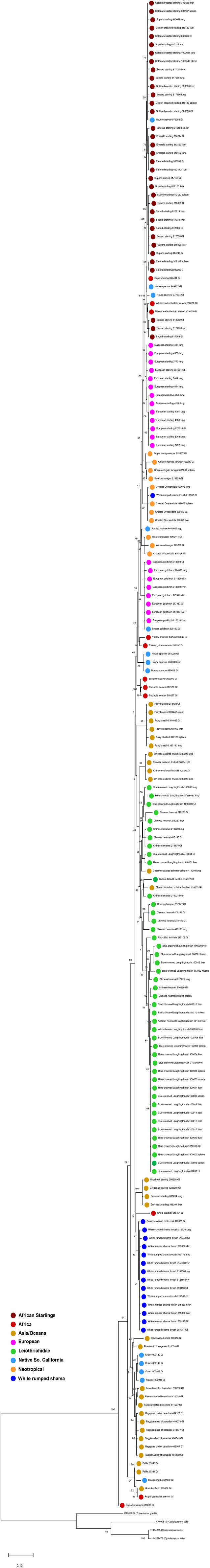
Phylogenetic analysis of 181 partial amino acid sequences of the *Isospora* COI gene isolated from passerine birds. Sequences represent a monophyletic group with lineages grouping by host species. COI phylogeny inferred by using the maximum likelihood method and Tamura–Nei model conducted in MEGA. The tree with the highest log likelihood (−718.29) is shown. The percentage of trees in which the associated taxa clustered together is shown next to the branches. A discrete Gamma distribution was used to model evolutionary rate differences among sites [five categories (+G, parameter = 0.5511)]. The tree is drawn to scale, with branch lengths measured in the number of substitutions per site. COI gene of *Toxoplasma gondii* used as an outgroup. Trees generated with 500 bootstrap replicates.

The topology of the phylogenetic tree based on *Isospora* sp. COI sequences demonstrates grouping of lineages by avian host species and continent of origin (Figure 1, Supplementary Figure 1). The largest lineage in the tree was composed of a clade of 54 sequences, with 48 of them associated with starlings: a subclade entirely of European starling tissues [New World, US (introduced)] and smaller subclades of African starling tissues including superb, emerald, and golden-breasted starlings. The non-starling sequences also came from three additional African species (a southern cape sparrow and two white-headed buffalo weavers) and three house sparrows (New World, US). Other clades in this large lineage were also fairly host-specific as listed as follows: One cluster of sequences from three sociable weavers (Old World, Africa); one with three house sparrows; one with eight sequences from European goldfinch (Old world, Europe) and one lesser goldfinch (New World, US); and finally, branches representing neotropical bird species including crested oropendola, purple honeycreeper, and tanagers.

There was another large of host-specific parasites, with a clade almost entirely from tissues of birds of the family Leiothrichidae—the laughingthrushes, Chinese hwamei, leiothrix, and liocichla—and smaller clades represented by other Asian species, the Chinese collared finchbill and Fairy bluebird. Sequence identity was variable among *Isospora* sp. sequences from Leiothrichidae, with a large group of 29 sequences ranged 96.6%−100% with an average of 98.7%, while COI sequence identity from all birds in this family (45 sequences) ranged 94.2%−100% with an average of 97.1%.

Additional lineages had *Isospora* sp. sequences that clustered by a single or closely related avian host species including white-rumped shama (Old World; India/SE Asia), Palila (Hawaii), Raggiana bird of paradise and fawn-breasted bowerbirds (Old World; New Guinea), Corvids (crow/raven, New World, US), and Grosbeak starling (Old World, Indonesia).

Exceptions to grouping of *Isospora* sp. COI sequences by host existed throughout the tree. One sociable weaver was distinct from a group of three others with 94.2% average identity compared to 99.3% among the three other sociable weavers. While all 13 COI sequences from seven Chinese hwamei were within the Leiothrichidae clade, they consisted of multiple distinct clusters. Among COI sequences from this species, the average sequence identity was 96.4%, and the majority of pairwise identities (60 out of 78) were below 97%. Two pairs of sequences from blue-crowned laughingthrush (also family Leiothrichidae) were also divergent from the larger group, with as low as 94.2% identity to the rest which shared an average of 97.9% identity. Further, the divergent sequences occurred among different tissues form the same blue-crowned laughingthrush; liver and gastrointestinal (GI) from one bird were 99.5% identical, while its lung sample was 97.3% and 97.2% identical to the liver and GI sequence, respectively.

Among the 14 sequences from eight white-rumped shamas, one sequence was divergent with as low as 95.1% identity compared to all 13 other more closely related sequences which ranged from 98.4%−100% with a mean of 99.4%. The divergent sequence clustered with tissues from a New World, neotropical species, the crested oropendola. One house sparrow sequence grouped with a subclade of 12 African starlings, and two others in a small subclade with a southern cape sparrow. Other sequences representing single branches of infected tissues from birds whose *Isospora* sequences did not cluster by host species included those obtained from African birds (sociable weaver, purple grenadier, and oriole warbler), Native US birds (mockingbird and lesser goldfinch), and Asian/oceanic birds (chestnut-backed scimitar babbler and Gouldian finch).

## Discussion

The results of this study are part of a larger investigation regarding *Isospora* sp. infections in mixed-species aviaries. The current data aimed to demonstrate host-specificity of *Isospora* sp. parasites in passerine birds by sequencing parasites obtained from a large number of diverse avian hosts. Given the diversity of host species and the ubiquity of *Isospora* spp. identified in passerine birds, this gene was chosen as an appropriate target for detecting coccidia and resolving their species.

These data demonstrated a monophyletic group of *Isospora* spp. based on partial sequencing of the COI gene. Similar to previous work with fewer birds, the data show a high degree of genetic diversity with almost as many unique genotypes as there were total sequences. However, the topology of the COI tree reinforces the host-specificity for *Isospora* spp. detected, exemplified by host species from geographically distinct regions harboring parasites of highest genetic similarity. For example, the blue-crowned laughingthrush with samples from both San Diego and Europe, as well as European starlings made up of individuals from San Diego and a wild-caught group from the San Francisco Bay.

The exceptions to grouping of *Isospora* sp. sequences by avian hosts could represent expanding host range for certain parasites that have species-jumped opportunistically. While co-phylogeny has been demonstrated with multiple species of birds and their respective coccidia (2), in other *Eimeria* it is suggested that host-adaptation rather than co-phylogeny explains incongruencies between hosts and their coccidia (15, 20). True host-switching with adaptation to a new host after a species-jumping event represents a complex host–parasite relationship dependent on multiple factors over time (9, 10). Determining whether outliers and multiple genotypes in a single avian species are spillover events or true host-switches is not clear from this snapshot of sequencing data. However, it has been proposed that birds harboring more than one *Isospora* spp. simultaneously may be common in the wild (2, 21). Therefore, large aviaries with breeding colonies establishing generations of hosts with diverse and non-sympatric natural histories may allow for repeated exposure to new parasites. In these unique environments, it is important to note the possibility of pseudoparasitism, where environmental oocysts shed by other avian species are ingested and excreted having passed-through the GI tract without infection or replication.

Intra-species diversity demonstrated with single outliers (white-rumped shama) or multiple groups of outliers (Chinese hwamei and Leiothrichidae species of blue-crowned laughingthrush and scarlet-faced liocichla) may support the idea that some *Isospora* spp. or certain avian hosts experience *Isospora* spp. species-jumping. In addition to numerous clades of single or closely related species, there was a pattern for groups with different host species to be grouped by continent of origin, and a distinction between groups of New World birds compared to Old World. Another example is seen in the grosbeak starling (Old World, Indonesia), which is a member of the family Sturnidae as are all other starlings. However, the grosbeak is the only Asian starling in this tree, with *Isospora* sequences divergent from those from African starlings while sharing highest genetic relatedness to white-rumped shama parasites, another bird with Southeast Asian origins. Exceptions to this were seen as well, highlighted when there were very few members of a certain avian host. For example, an *Isospora* sp. from a purple grenadier (Africa) had highest identity to parasites from a Gouldian finch (Oceana), followed by a mockingbird (So. California) and Palila (Oceana). Similarly, house sparrow parasites (invasive US species originating from Asia and Europe) clustered with those from African species such as the starlings, Southern cape sparrow, golden weaver and yellow-crowned bishop.

It has been suggested that parasite species with life cycles restricted to the GI tract are host-specific, but those that have asexual, replicating forms in mononuclear cells as part of their life cycle have unknown host-specificity (22). Experimental inoculation of different passerine species with tissue homogenates containing circulating asexual stages resulted in infection of multiple different passerines, but when oocysts were used, only conspecifics were able to be infected (23, 24). It could be that parasites with circulating stages are able to jump species and therefore eventually achieve true host-switching more efficiently. Experimental infections are likely necessary to show how readily birds can become infected with different parasites and whether differences in host-specificity are dependent on parasite life cycle. This would also help clarify whether outliers identified in these studies represent pass through of ingested oocysts rather than true parasitism. Furthermore, concrete genetic distinctions would need to be established to determine how much divergence represents a distinct *Isospora* spp. It has been proposed based on other gene targets that 1% divergence could indicate a different *Isospora* spp.; however, that delineation is not clear for COI gene, and should be combined with morphologic and ultrastructural analysis to be conclusive. As the COI assay is reliable in detecting a diverse range of *Isospora* sp. sequences, rigorous analysis of host factors, disease outcome, and epidemiology combined with the genetic data can provide valuable insight into the occurrence of true species-jumping and host-switching, as well as the disease consequences of those events. With concerns for host-switching hindering conservation goals of captive breeding and releasing, this database of genetic sequences will facilitate conservation program management and disease risk analyses for breeding, reintroduction, and translocation.

## Data Availability Statement

The datasets presented in this study can be found in online repositories. The names of the repository/repositories and accession number(s) can be found at: https://www.ncbi.nlm.nih.gov/genbank/, OL999103–OL999283.

## Ethics Statement

The animal study was reviewed and approved by Samples tested from San Diego Zoo Wildlife Alliance fall under an approved biomaterials request for non-invasive samples and IACUC protocol #18-024 and #21-019 “Opportunistic Sampling During Veterinary Procedures and Husbandry Procedures.” Stanford biosamples were collected as part of a diagnostic work-up conducted by a clinical veterinarian. These animals were being captured and housed for flight-related research under an animal care and use protocol approved by Stanford's Institutional Animal Care and Use Committee (IACUC).

## Author Contributions

SK: study design, data analysis, manuscript writing, and editing. BR: study design and manuscript editing. CW: study design, manuscript editing, data analysis, and preparation. JB: data acquisition and analysis, manuscript editing, and preparation. DC and AZ: data acquisition and analysis and manuscript editing. AB, JV-M, and SF: data acquisition and manuscript editing. All authors contributed to the article and approved the submitted version.

## Funding

This project was funded by a generous philanthropic grant from the McBeth Foundation (internal ID 32121-McBeth/Avian *Isospora*).

## Conflict of Interest

The authors declare that the research was conducted in the absence of any commercial or financial relationships that could be construed as a potential conflict of interest.

## Publisher's Note

All claims expressed in this article are solely those of the authors and do not necessarily represent those of their affiliated organizations, or those of the publisher, the editors and the reviewers. Any product that may be evaluated in this article, or claim that may be made by its manufacturer, is not guaranteed or endorsed by the publisher.
