# Supplementary figures and images for: Mitochondrial Gene Diversity and Host Specificity of Isospora in Passerine Birds
Source: Front Vet Sci. 2022 Jun 29;9:847030. doi: 10.3389/fvets.2022.847030 (PMC9280662; doi:10.3389/fvets.2022.847030)

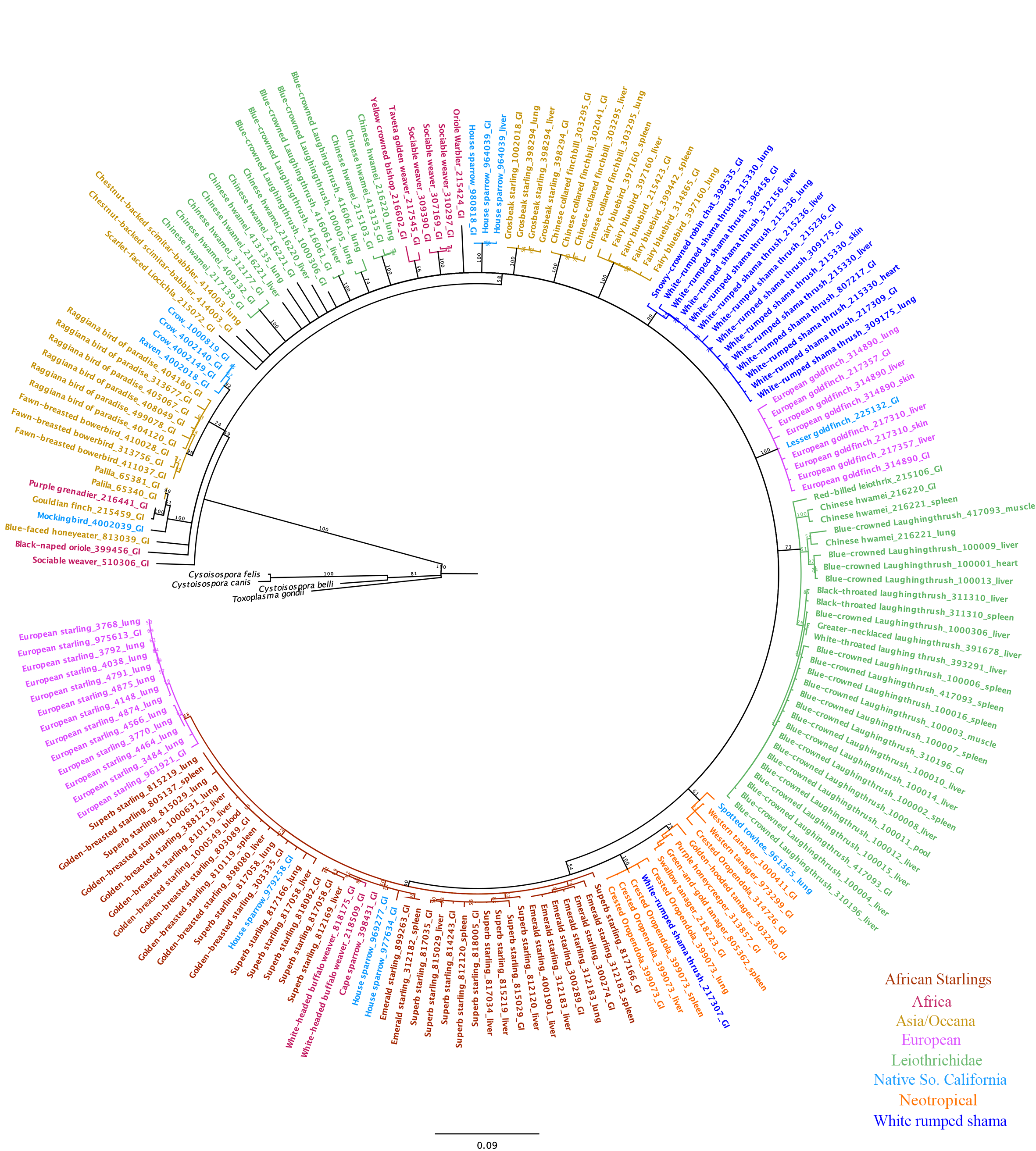

Supplement: Supplementary file 2 [file Image_1.TIF]
